# Mitigating Computer Limitations in Replicating Numerical Simulations of a Neural Network Model With Hodgkin-Huxley-Type Neurons

**DOI:** 10.3389/fninf.2022.874234

**Published:** 2022-05-12

**Authors:** Paulo H. Lopes, Bruno Cruz Oliveira, Anderson Abner de S. Souza, Wilfredo Blanco

**Affiliations:** ^1^Bioinformatics Department, Federal University of Rio Grande do Norte, Natal, Brazil; ^2^Computer Science Department, State University of Rio Grande do Norte, Natal, Brazil

**Keywords:** neural network, Hodgkin-Huxley-type neurons, numerical replicability, floating-point precision, code refactorization, computer languages

## Abstract

Computational experiments have been very important to numerically simulate real phenomena in several areas. Many studies in computational biology discuss the necessity to obtain numerical replicability to accomplish new investigations. However, even following well-established rules in the literature, numerical replicability is unsuccessful when it takes the computer's limitations for representing real numbers into consideration. In this study, we used a previous published recurrent network model composed by Hodgkin-Huxley-type neurons to simulate the neural activity during development. The original source code in C/C++ was carefully refactored to mitigate the lack of replicability; moreover, it was re-implemented to other programming languages/software (XPP/XPPAUT, Python and Matlab) and executed under two operating systems (Windows and Linux). The commutation and association of the input current values during the summation of the pre-synaptic activity were also analyzed. A total of 72 simulations which must obtain the same result were executed to cover these scenarios. The results were replicated when the high floating-point precision (supplied by third-party libraries) was used. However, using the default floating-point precision type, none of the results were replicated when compared with previous results. Several new procedures were proposed during the source code refactorization; they allowed replicating only a few scenarios, regardless of the language and operating system. Thus, the generated computational “errors” were the same. Even using a simple computational model, the numerical replicability was very difficult to be achieved, requiring people with computational expertise to be performed. After all, the research community must be aware that conducting analyses with numerical simulations that use real number operations can lead to different conclusions.

## Introduction

A fundamental point to the evolution of science is the possibility of analyzing previous studies, and from that to propose improvements, reflections or new conclusions. A scientific experiment or analysis should ideally be described in sufficient detail so that other scientists with sufficient skills and means can follow the steps described in published work and obtain the same results within the margins of experimental error (Plesser, 2018).

However, such attention to experimental error must be increased when scientists use digital computers to perform experimental simulations and data analyses. It is possible to perceive researchers assuming that results obtained by computers could be trusted in many investigations, provided that the principal algorithms and methods employed were suitable to the problem at hand. Little or no attention is paid to the correctness of implementation, the potential sources for error, or the influences introduced by system software and hardware; in addition, how difficult it can be to replicate a computational experiment after some years or even weeks (Plesser, 2018; National Academies of Sciences Medicine, 2019).

These aspects fall into the two terminologies of reproducibility and replicability, which are recurrent topics in science, specifically in a computational context in which results/data are produced by computational simulation programs, usually developed using source code in a specific computer language. This recurrence mainly starts from the difficulty of reusing codes, modules and functions developed in previous studies. In the context of the computational programs, the Association for Computing Machinery (ACM) proposes the following definitions (https://www.acm.org/publications/policies/artifact-review-and-badging-current), which are adopted in this manuscript:

**Replicability** (Different team, same experimental setup): The measurement can be obtained with stated precision by a different team using the same measurement procedure, the same measuring system, under the same operating conditions, in the same or a different location on multiple trials. For computational experiments, this means that an independent group can obtain the same result using the author's own artifacts.

**Reproducibility** (Different team, different experimental setup): The measurement can be obtained with stated precision by a different team, a different measuring system, in a different location on multiple trials. For computational experiments, this means that an independent group can obtain the same result using artifacts which they develop completely independently.

It is important to mention that there are different definitions for these terms (Plesser, 2018), including inverse definitions in relation to those adopted in this study, as proposed by Claerbout and Karrenbach (1992) and Rougier et al. (2017). These two aspects are considered fundamental for a scientific software program to be presented as a scientific contribution (Benureau and Rougier, 2018). In this sense, several recommendations have already been suggested to achieve computational reproducibility and/or replicability of numerical results obtained by computational models (Sandve et al., 2013; Benureau and Rougier, 2018; Elofsson et al., 2019).

Let us think hypothetically of a scenario where we have access to the entire source code and a precise description of “*all*” the documentation and steps to re-execute the code under a specific computational environment (ex: 32/64 hardware architecture, Compilers/Interpreters versions and libraries). Other scientists should preferably be willing to follow the described steps and obtain the same results (Plesser, 2018). However, the code usually needs to be executed in different operating systems (OS) and hardware platforms (HPs). A previous work showed that numerical replicability was not achieved under these conditions (Blanco et al., 2020), questioning whether the efforts of well-established international standards (IEEE, 2019) are followed among programming languages and their implementations, libraries are being used (with their respective compiler and compilation options) and HPs.

The representation of real numbers by computers is an intrinsic source of error for numerical simulations. As it is known, data is stored on computers in binary format. Hence, each different type of data requires a different representation form. In the case of real numbers, there are a variety of forms of different representations, with the IEEE 754 standard being the most widely used in modern computers. Regardless of the standard used, all representation of real numbers in binary is a discretization of an infinite space; therefore, representation errors are generated (Franco et al., 2017). Since there is no exact representation for all floating-point (FP) numbers after mathematical operations, including the initializing ones (first time a value is assigned to a variable), rounding-off and truncation operations are necessary to find the closest representation to the desired value. Summatory operations are a classic example in which numbers are rounded at each step, therefore they are non-commutative and non-associative. For example:

Commutative:


$$\begin{array}{l} \{\begin{array}{c} \begin{array}{c} -0.3+0.1+0.1+0.1=0.0000000000000000277556 \end{array}\\ \begin{array}{c} 0.1+0.1+0.1-0.3=0.0000000000000000555112 \end{array} \end{array} \end{array}$$

Associative:


$$\begin{array}{l} \{\begin{array}{c} \begin{array}{c} 0.1+0.1+0.1-0.3=0.0000000000000000555112 \end{array}\\ \begin{array}{c} 0.1+0.1+\left(0.1-0.3\right)=0.0000000000000000277556 \end{array} \end{array} \end{array}$$

Almost all the results remained imprecise even using well-known available methods which were developed to reduce the errors in operations using floating-point numbers. For example, when the Kahan summation (Kahan, 1965) method was used, only two of the previous examples were correctly solved. On the other hand, the ReproBLAS (Demmel et al., 2016) and ExBLAS (Iakymchuk et al., 2015) showed to be unsusceptible, as they generated the same errors; however, they still failed to produce the correct result.

Kahan Commutative:


$$\begin{array}{l} \{\begin{array}{l} \begin{array}{c} -0.3+0.1+0.1+0.1=0.0000000000000000277556 \end{array}\\ 0.1+0.1+0.1-0.3=\;0 \end{array} \end{array}$$

Kahan Associative:


$$\begin{array}{l} \{\begin{array}{l} 0.1+0.1+0.1-0.3=\;0\\ \begin{array}{c} 0.1+0.1+\left(0.1-0.3\right)=0.0000000000000000277556 \end{array} \end{array} \end{array}$$

ExBLAS/ReproBLAS Commutative:


$$\begin{array}{l} \{\begin{array}{c} \begin{array}{c} -0.3+0.1+0.1+0.1=0.0000000000000000277556 \end{array}\\ \begin{array}{c} 0.1+0.1+0.1-0.3=0.0000000000000000277556 \end{array} \end{array} \end{array}$$

ExBLAS/ReproBLAS Associative:


$$\begin{array}{l} \{\begin{array}{c} \begin{array}{c} 0.1+0.1+0.1-0.3=0.0000000000000000277556 \end{array}\\ \begin{array}{c} 0.1+0.1+\left(0.1-0.3\right)=0.0000000000000000277556 \end{array} \end{array} \end{array}$$

As shown, this lack of precision already directly impacts simple operations; therefore, they will not be applied in our network case, which is a more complex implementation.

This study address how summation operations performed under real numbers evolve to non-replicability in numerical computations. To achieve this goal, we used a computational simulation that represents the activity of a neural network composed by the Hodgkin-Huxley (HH) neuron model. Using the default floating-point precision (DFPP—double type for C program language), our previous work proved that different associations of pre-synaptic current values during the summation of the pre-synaptic activity affected numerical replicability (Blanco et al., 2020). This study is going one step further in attempting to mitigate previously reported non-replicability issues, but now also including the commutation of the pre-synaptic currents. Moreover, the original model implemented in C/C++ was refactored and ported to another 3 programming languages/software widely used in scientific computing: XPP/XPPAUT, Python, and Matlab. Simulations were also executed under Windows and Ubuntu Linux OSs. The question to be answered is: Will the replicability be maintained across computer languages and OSs? Thus, over 72 simulations were executed to cover these aspects.

## Materials and Methods

### Neural Network Model

Early studies already modeled the spontaneous neural network activity during early development, which is characterized by a cyclic profile where episodes of strong neural activity are followed by episodes of quiescence (Tabak et al., 2010; Blanco et al., 2017). This pattern had been seen in regions such as: the spinal cord (Tabak et al., 2001), retina (Grzywacz and Sernagor, 2000) and cortical networks (Opitz et al., 2002).

All simulations model 8 s of a small all-to-all connected neural network composed by 100 Hodgkin-Huxley neurons. Each *j* neuron is modeled by two ordinary differential equations (ODE), the membrane potential (Equation 1) and the fraction of activated delayed rectifier K^+^ channels (Equation 2).


(1) $$\begin{array}{l} C\frac{dV_{j}}{dt}=-\left[I_{Na_{j}}+I_{K_{j}}+I_{l_{j}}+I_{syn,e_{j}}+I_{syn,i_{j}}-I_{app}\right] \end{array}$$

(2) $$\begin{array}{l} \frac{dn_{j}}{dt}=\alpha_{n}\left(V_{j}\right)\left(1-n_{j}\right)-\beta_{n}\left(V_{j}\right)n_{j} \end{array}$$

(3) $$\begin{array}{l} a_{n}\left(V\right)=\frac{0.01\left(10.0-V\right)}{\left(e^{0.1\left(10.0-V\right)}-1.0\right)} \end{array}$$

(4) $$\begin{array}{l} \beta_{n}\left(V\right)=0.125e^{-V/80} \end{array}$$

The membrane potential equation integrates several currents. The Na^+^ (*I*_*N*_*a*__*j*__) current assumes instantaneous activation (Rinzel, 1985) (Equation 5). The K (*I*_*K*_*j*__) and the leakage (*I*_*l*_*j*__) currents are represented in Equations (6) and (7), respectively. The synaptic currents, coming from a *N*_*e*_ sub-population of excitatory neurons and *N*_*i*_ remaining inhibitory neurons, are presented in Equations (8) and (9), respectively. Their corresponding synaptic conductance are shown in Equations (10) and (11).


(5) $$\begin{array}{l} I_{Na}=g_{Na}m_{\infty }^{3}\left(V_{j}\right)\left(0.8-n_{j}\right)\left(V_{j}-V_{Na}\right) \end{array}$$

(6) $$\begin{array}{l} I_{K_{j}}={g_{K}n}_{j}^{4}\left(V_{j}-V_{K}\right) \end{array}$$

(7) $$\begin{array}{l} I_{l_{j}}=g_{l}\left(V_{j}-V_{l}\right) \end{array}$$

(8) $$\begin{array}{l} I_{syn,e_{j}}=g_{syn,e_{j}}\left(V_{j}-V_{exc}\right) \end{array}$$

(9) $$\begin{array}{l} I_{syn,i_{j}}=g_{syn,i_{j}}\left(V_{j}-V_{inh}\right) \end{array}$$

(10) $$\begin{array}{l} g_{syn,e_{k}}=\frac{{\bar{g}}_{syn}}{N}\overset{N_{e}}{\underset{j=1}{\sum }}a_{j}s_{j}-a_{k}s_{k} \end{array}$$

(11) $$\begin{array}{l} g_{syn,i_{k}}=\frac{{\bar{g}}_{syn}}{N}\overset{N}{\underset{j=Ne+1}{\sum }}a_{j}s_{j}-a_{k}s_{k} \end{array}$$

The input current *I*_*app*_ in Equation (1) guaranties the diversity of activity in the population, since different current input values were assigned for each neuron. The vector of *I*_*app*_ values was randomly generated from a uniform distribution over the range −10 to 5$\frac{\mu A}{cm^{2}}$ and maintains its values for all simulations.

Two variables are the main outputs for each neuron, namely the spontaneous activity, and the synaptic efficacy, represented by *a*_*j*_ and *s*_*j*_, respectively. These two variables present a cyclic profile; *a*_*j*_ has a fast profile variable with elevated activity episodes divided by quiescent periods (inter-episode intervals), while *s*_*j*_ is a slow profile during quiescent periods of *a*_*j*_, but has fast depression during the high activity episodes (Tabak et al., 2010; Blanco et al., 2017). Their equations have the form:


(12) $$\begin{array}{l} \frac{da_{j}}{dt}=\Pi \left(V_{j}\right)\alpha_{a}\left(1-a_{j}\right)-\beta_{a}a_{j} \end{array}$$

(13) $$\begin{array}{l} \frac{ds_{j}}{dt}=\alpha_{s}\left(1-s_{j}\right)-\Pi \left(V_{j}\right)\beta_{s}s_{j} \end{array}$$

In which: $\Pi \left(V_{j}\right)=1/\left(1+e^{\left(v_{th}-V_{j}\right)/kv_{j}}\right)$ reflects synaptic release when the presynaptic voltage *V*_*j*_ depolarizes above *V*_*th*_ during an action potential. The average network activity and synaptic efficacy are <A> $=\frac{1}{N}\sum_{j=1}^{N}a_{j}$ and <S> $=\frac{1}{N}\sum_{j-1}^{N}s_{j}$, respectively. The network model parameters are shown in Table 1.

**Table 1:** Parameters of the network model using Hodgkin-Huxley-type neurons.

**Parameter**	**Description**	**Value**
*g_*l*_*	Leak conductance	0.1 S/cm^2^
*V_*l*_*	Leak reversal potential	−10.6 mV
*g_*Na*_*	Sodium conductance	36 mS/cm^2^
*V_*Na*_*	Sodium reversal potential	115 mV
*g_*k*_*	Potassium conductance	12 mS/cm^2^
*V_*k*_*	Potassium reversal potential	−12 mV
* ${\bar{g}}_{syn}$ *	Max. synaptic conductance	3.6 mS/cm^2^
*V_*exc*_*	Excitatory reversal potential	70 mV
*V_*inh*_*	Inhibitory reversal potential	70 mV
*I_*app*_*	Input or applied current	−10 to 5 μA/cm^2^
*α_*a*_*	Synaptic activation rate	1 ms^−1^
*β_*a*_*	Synaptic decay rate	0.1 ms^−1^
*α_*s*_*	Synaptic recovery rate	0.0015 ms^−1^
*β_*s*_*	Synaptic depression rate	0.12 ms^−1^
*V_*th*_*	Threshold for activation/depression	40 mV

Excitatory and inhibitory neurons were modeled using the same equations, however changing their reversal potential value (*V*_*exc*_ and *V*_*inh*_ in Equations 8, 9 and values from Table 1). Although *V*_*exc*_ = *V*_*inh*_ = *70 mV* for all simulations, this configuration set the inhibitory neurons to behave like excitatory ones. In other words, they are just labeled as inhibitory in simulations where inhibitory set of neurons are defined.

**Figure 1:**
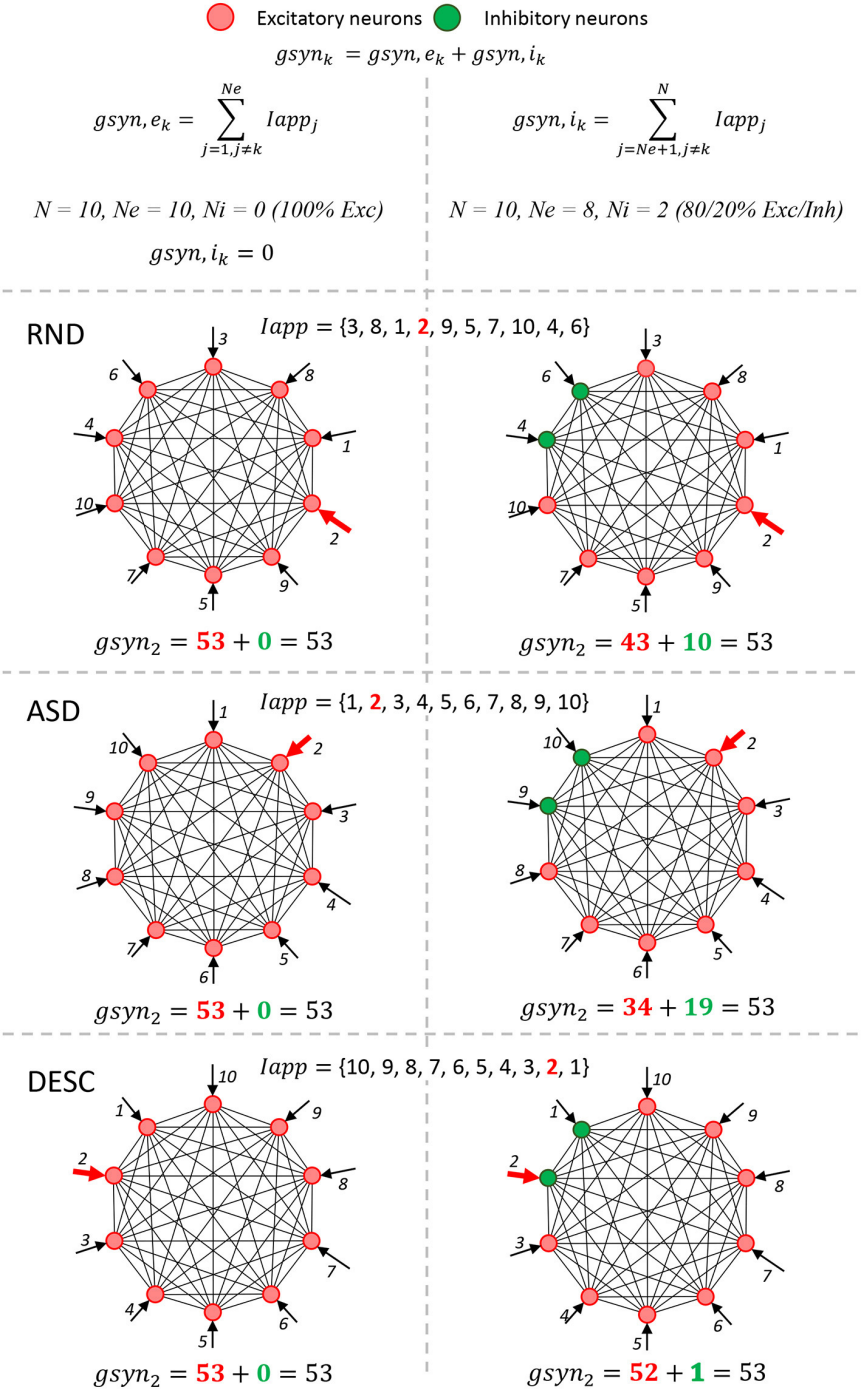
The integration of pre-synaptic activity must obtain the same value. A simple neural network schema composed by 10 neurons shows the six simulation scenarios (3x2, rows and columns) that modify the computational model (Table 2, fourth and fifth rows). The columns represent two scenarios involving the proportion of ***Ne*** and ***Ni***: 100% excitatory neurons (first column) and 80/20% of excitatory/inhibitory neurons (second column). These scenarios change the associativity in the conductance summation of presynaptic activity values (Equations 10 and 11). For simplification, the formulas and graphs represent the integration of presynaptic activity of one neuron *k* (*k*=*2*,*g*_*syn*2_), and the *Iapp* values were integers (also similar to indices). When 100% of neurons are excitatory, only one summation was calculated (*g*_*syn*,_*e*__*k*__), since there are no inhibitory neurons in the network (*g*_*syn*,_*i*__*k*__= 0); while in the 80/20% case, the total *g*_*synk*_ is calculated with two summations, the *g*_*syn*,_*e*__*k*__ and *g*_*syn*,_*i*__*k*__. Rows from the second to the end represent three ways of how the *I*_*app*_ vector is ordered: Random (RND), Ascendant (ASD) and Descendent (DESC). These scenarios change the commutation in the conductance summation of presynaptic activity values. The total summation formulas for all scenarios are shown at the bottom of each cell.

A special attention should be given to three parameters: input current *I*_*app*_, amount of excitatory neurons *N*_*e*_, and the amount of inhibitory neurons *N*_*i*_. Their manipulation defines the numerical simulations scenarios presented in this study (see next sections and Figure 1).

### Simulation Scenarios

The simulation scenarios proposed in this study are presented in Table 2. The simulations were executed on the same hardware platform: Intel i5-9600k 3.70GHz 8GB DDR4. However, it was Dual Boot configured with Windows and Ubuntu Linux operating systems (OSs) (Table 2, first row). The same versions of the C/C++ (https://isocpp.org/), XPP/XPPAUT (Ermentrout and Simulating, 2002), Python (https://www.python.org/), and Matlab (https://www.mathworks.com/) languages/software programs were installed in both OSs (Table 2, second row). The model implemented in C/C++ (Blanco et al., 2020) was carefully refactored to a new version, allowing the model (network structure, initial conditions, parameters values and ODEs) to be easily transcripted into the proposed programing languages/software programs. Simulations were executed using two floating-point precision types: the default double floating-point precision defined by IEEE 64 (IEEE, 2019) (referred to throughout the text as DFPP) and an implemented high floating-point precision type (referred to throughout the text as HFPP). The parameters presented so far do not alter the neural network model.

**Table 2:** Simulation parameters.

**Simulation parameters**		**Values**	**Description**
Operating system	OS	WIN LIN	Windows 10 Pro Ubuntu Linux 20.04 LTS
Programing Languages/Software	PL	XPP/XPPAUT C/C++ Python Matlab	XPP/XPPAUT version 6.9.0.31 GCC/G++ version 9.3.0 Python version 3.8.0 Matlab version 9.5.0
Floating-point Precision	FPP	DFPP HFPP	Default Floating-point precision High Floating-point precision
***N**_***e***_* and ***N**_***i***_* proportions	SPLT	100 80/20	100% excitatory neurons (no association) 80% excitatory and 20% inhibitory neurons
***I**_***app***_* commutation	SORT	RND ASD DESC	Random organization Ascendent organization Descendant organization

Similar to the study by Blanco et al. (2020), there are two main scenarios regarding the proportion of ***N***_***e***_ and ***N***_***i***_: (1) A network composed by only excitatory neurons (Table 2, fourth row, value 100—no association); and (2) a network composed by 80% excitatory and 20% fake inhibitory neurons (reversal potential *V*_*exc*_ = *V*_*inh*_ = *70 mV*, hence, they are still playing an excitatory role) (Table 2, fourth row, value 80/20). In the first case, the integration of presynaptic activity conductance is not split (Figure 1, first column, 100% *Exc* and *IappInhi* = 0). However, the integration of presynaptic activity is split in the second case (Figure 1, second column, 80/20% *Exc*/*Inh*).

Furthermore, we also tested whether the order (commutating the values) of the *I*_*app*_ vector could affect the simulation results. To do so, three new scenarios were tested: (1) *Random*, in which the values were randomly generated once (Table 2, fifth row, value RND; and Figure 1, RND row); (2) *Ascendant*, in which the same values generated in (1), were sorted upwardly (Table 2, fifth row, value ASD; and Figure 1, ASD row); and (3) *Descendant*, in which the same values generated in (1) were sorted downwardly (Table 2, fifth row, value DESC; and Figure 1, DESC row).

Thus, a total of 92 scenarios were proposed from Table 2. However, we could not execute the HFPP under XPP/XPPAUT and Matlab, ending with a total of 72 computational simulations. It is important to mention that all of the 72 proposed simulations should obtain identical values of <A> and <S> over time; in other words, replicability should be achieved even when the model parameters are changed (proportion of ***N***_***e***_ and ***N***_***i***_, and the commutation of *I*_*app*_ vector values) (Figure 1). In cases in which <A> and <S> keep the cyclic profile, but not necessarily the same values, it is considered that reproducibility was achieved.

The simulations using HFPP were our gold standard result, and were used to compare the rest of the simulations. The temporal discrepancies between the average spontaneous activity <A> of two simulations *i* and *j* will be calculated using the absolute error equation ϵ (t) = |A_i_ (t) − A_j_ (t)|.

### Implementation in Other Languages/Software Programs

The source code originally implemented in C/C++ and published in Blanco et al. (2020), went through a refactoring process to further facilitate manual conversion from C/C++ code to the target languages/software programs: Python, and Matlab. Specific syntax elements of the target's languages were carefully incorporated during this transcription to maintain the similarity among the source codes as much as possible, while also taking advantage of the particularities that each language offers.

We also implemented the original code into the XPP/XPPAUT tool. Its script language is not considered a native computer language, but it is widely used in the field of scientific computation, and known for facilitating high-level implementation, and consequently speeding up the production of results.

Following the suggestions by Sandve et al. (2013) and Elofsson et al. (2019), the codes were implemented and maintained the same organization in terms of initialization, nomenclature, manipulation of variables, functions, files, and directories. Hence, the variables, functions and comments were positioned in the same line of each source code. Moreover, Python and Matlab source code files contain several blank lines to match the larger specification syntax usually required by the C/C++ implementations.

Programing languages have different nomenclatures/names for the same data types. This is the case when a variable of 64 bits of floating-point precision is used. For example, they are declared as “double” in C/C++ and Matlab; however, they are “float” in Python (Table 3, first row). Only the C/C++ and Python source code were adapted to run simulations using HFPP types, which are implemented from third-party libraries (Table 3, second row). Hence, the files have additional lines for initializing variables. Boost multiprecision library with cpp_dec_float_100 backend was used in C/C++. It guaranties 100 accurate digits (https://www.boost.org/doc/libs/1_78_0/libs/multiprecision/doc/html/boost_multiprecision/tut/floats/cpp_dec_float.html). Decimal class was used in python, it stores how many digits are assigned to the object during its creation. The precision was defined in context [*getcontext().prec* =*100*] and it is only used during arithmetic operations (https://docs.python.org/3/library/decimal.html). Although Matlab offers a way to implement HFPP through Variable-precision arithmetic (VPA) type from Symbolic Math Toolbox, the results were not presented in this study because its execution is extremely time-consuming (to the order of months).

**Table 3:** Floating-point precision (FPP) digits and types used in each language.

**Languages/software**	**DFPP type/digits**	**HFPP type/digits**
XPP/XPPAUT	Double/8	-
C/C++	Double/15	Boost/100
Python	Float/15	Decimal/100
Matlab	Double/15	-

ODEs were solved by Runge-Kutta fourth order (RK4) method. Implementations for C/C++, Python and Matlab were taken from https://people.sc.fsu.edu/~jburkardt/cpp_src/rk4/rk4.html. The all-source code of this study is available as freeware in the Github repository at the hyperlink https://github.com/wblancof/neural-numerical-replicability2.git.

The main abbreviations utilized in this work are available in Table 4.

**Table 4:** Abbreviation table.

**Abbreviations**	**Definitions**
ACM	Association for Computing Machinery
ASD	Ascendant sorted
DESC	Descendant sorted
DFPP	Default floating-point precision
FP	Floating-point
HFPP	High floating-point precision
HH	Hodgkin-Huxley
HP	Hardware platforms
IEEE	Institute of Electrical and Electronics Engineers
ODE	Ordinary differential equations
OS	Operating systems
RK4	Runge-Kutta fourth order
RND	Random sorted
VPA	Variable-precision arithmetic

## Results

This section shows the numerical results from the computational simulations proposed in Table 2. The simulations model the activity of a population of neurons during early development. It is necessary to note that all the simulations must theoretically obtain identical values for the spontaneous network activity <A> (Equation 12) and the network synaptic efficacy <S> (Equation 13) over time.

The execution simulation times are presented in Table 5. The simulations that used HFPP were the most time-consuming as expected, with the Python language on Windows being the most time-consuming in taking approximately 49 h. On the other hand, the simulations that used DFPP were the least time-consuming, with C++ language on Ubuntu Linux only taking about 34 s. Next, we decided to divide the simulation results into two main categories to better organize the Results Section: High floating-point precision (HFPP) and Default floating-point precision (DFPP). This will help to compare the results more fairly and logically. We considered that XPP/XPPAUT simulations were under DFPP category; however, due to the lack of numerical precision of results and difficulty to control its high-level script language, it has its own sub-section.

**Table 5:** Time (in seconds) consumed for each computational simulation.

**Operating Systems →**	**Windows**					**Ubuntu Linux**			
**Precisions →**	**DFPP**		**HFPP**		**SORT**	**DFPP**		**HFPP**	
**Neurons proportions** **→** **Languages/Software** **↓**	**100**	**80/20**	**100**	**80/20**	**↓**	**100**	**80/20**	**100**	**80/20**
XPP/XPPAUT (Version 6.9.0.31)	477	464	**-**	**-**	RND	222	235	**-**	**-**
	471	471	**-**	**-**	ASD	222	236	**-**	**-**
	474	474	**-**	**-**	DESC	224	235	**-**	**-**
C/C++ (Version 9.3.0)	40	39	46,536	47,017	RND	34	34	35,663	34,947
	39	39	46,413	46,602	ASD	34	34	33,696	35,499
	39	39	46,550	47,425	DESC	35	34	35,250	35,784
Python (Version 3.8.0)	1,677	1,671	177,136	177,296	RND	1,014	1,033	103,349	103,730
	1,668	1,677	175,769	175,943	ASD	1,066	1,077	103,055	104,023
	1,665	1,668	177,611	177,859	DESC	1,073	1,080	103,431	103,476
Matlab (Version 9.5.0 2017b)	19,646	21,005	**-**	**-**	RND	13,447	13,450	**-**	**-**
	20,796	20,805	**-**	**-**	ASD	13,658	12,554	**-**	**-**
	20,417	20,457	**-**	**-**	DESC	13,164	12,982	**-**	**-**

### XPP/XPPAUT

**Figure 2:**
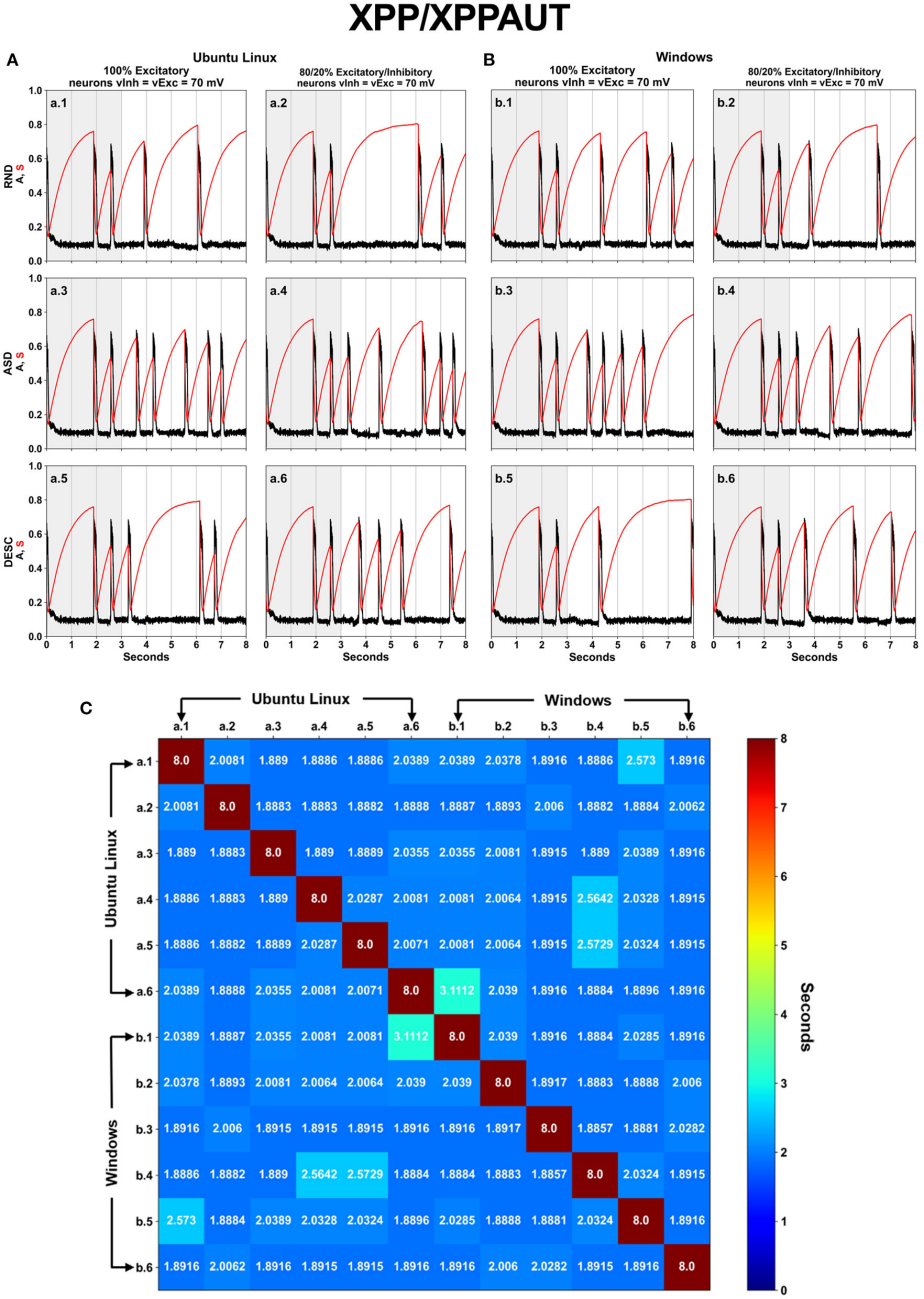
Associations and commutations of the *Iapp* vector produced different results on XPP/XPPAUT. The spontaneous network activity <A> (black curve) and the network synaptic efficacy <S> (red curve) are shown for a total of twelve (12) scenarios, which are the six (SPLT × SORT−2 × 3) scenarios shown in Figure 1 for Ubuntu Linux **(A)** and for Windows **(B)**. Panel **(C)** displays a square matrix with an all-to-all comparison of these 12 scenarios based on the absolute error between simulations. Each cell represents the first moment in time (seconds) when the absolute error reached a value >10^−4^.

Implementing the original code into XPP/XPPAUT script languages allowed us to easily test all proposed scenarios (Figure 1), with special attention to the *Iapp* vector order cases (Random, Ascendent, and Descendant) (Figure 1, rows), and constituting an aspect that was not explored in Blanco et al. (2020). We used the same parameters as in Tabak et al. (2010) and Blanco et al. (2020). The results confirmed the lack of numerical replicability between different OSs as shown in Blanco et al. (2020) (Figures 2A,B). Furthermore, we also confirmed that the order of *Iapp* vector is an issue which also affects the numerical replicability (Figure 2, rows).

The spontaneous network activity <A> and the network synaptic efficacy <S> display the cyclic patterns, however they are only visually similar until the second activity episode (Figure 2, time 0–3 s, highlighted with gray background). However, the numeric difference (>10^−4^) among the <A> profile from the simulations began in 2–3 s (Figure 2C). After the initial 3 s, remarkable differences are observed for all profiles of <A> and <S>. Nevertheless, there are three simulations which show similar results lasting for four seconds, including: Ubuntu Linux, 80/20, Ascendant sorted (Figure 2A-a.4); Ubuntu Linux 100 Descendant sorted (Figure 2A-a.5); and Windows 80/20 Ascendant (Figure 2B-b.4).

We found limitations with the XPP/XPPAUT tool. First, the maximum FPP allowed by the tool in the outputs is fixed up to eight decimal digits. This limitation did not allow us to compare the results with the other languages/software programs. Second, XPP/XPPAUT uses a high-level script language which is not capable to precisely initialize and manipulate variables, nor to control the source code flow. Third, we also noticed the necessity to access the source code of the solver method (RK4) to check functions eventually used in it, as well as possible truncation and rounding-off operations applied to floating-point numbers.

Due to these software limitations and the fact that we were unable to improve them (changing the XPP/XPPAUT source code and recompiling it are considered out of the scope of this study), these reasons guided us to not use other platforms or tools such as: Neuron (Hines and Carnevale, 1997), Brian (Stimberg et al., 2019), or NEST (Gewaltig and Diesmann, 2007). In this context, we decided to work on native programing languages which enable addressing these limitations.

### Code Refactoring to Mitigate the Lack of Replicability

The original C/C++ source code was refactored (source code taken from https://github.com/wblancof/neural-numerical-replicability). We must keep in mind that errors in representation, rounding-off and truncation operations are impossible to remove. Thus, we must learn to always generate the same “errors” regardless of the language, architecture and/or operating system used in order to improve the replicability of a code. To do this, we need to focus on two points: (1) use the same real number representation in all phases of a code; and (2) always perform operations in the same order. This section is dedicated to discussing a brief summary about these two points at different stages of a code.

#### Compilation/Interpretation

Modern compilers can make code optimizations to generate code that runs faster, and these optimizations can change the execution order of the instructions, generating different results according to the optimization used. In addition, compilers and interpreters perform conversions for intermediate representations which may result in different values (Boldo et al., 2015). One compiler can convert a literal real number to the single-precision (32-bit) floating-point representation in the IEEE 754 standard, while another can convert to the double-precision (64-bit) representation. Since not every number has an exact floating-point representation, this can generate different initialization values of the variables/parameters. For example, if a compiler converts literal real numbers from a code to single-precision representation, the value 0.1 is actually represented as 0.10000000149011611938. If the representation chosen is double precision, the value is now represented as 0.10000000000000000555.

We should use the same compiler version whether the code will be executed in different operating systems. However, the set of options used by the compiler may be different for different target architectures. Therefore, we must make sure that the floating-point representations used by the compiler/interpreter will be the same in different target architectures and/or operating systems. This task may be more difficult or impossible to be performed in the case of interpreted languages, since the interpreter can only be available in an executable form, meaning that it has already been compiled for different operating systems (Boldo et al., 2015).

#### Initialization

As we have already commented, rounding-off and truncation operations are performed after each floating-point arithmetic operation. Therefore, we must be careful when initializing real variables and constants, especially when porting code to another programming language. When the code was updated for the new version in C/C++ for this article and ported to XPP/XPPAUT, Python, and Matlab languages/software programs, we were careful to consider the different languages/software data types. Additionally, the code has to operate equally for DFPP and HFPP, and therefore mathematical operations in the initialization of variables were avoided. For example, the original initialization of the variable ${\bar{g}}_{syn}\;$was ${\bar{g}}_{syn}=3.6/nNeurons$, where *nNeurons* is a variable with the number of neurons (for the experiment, it was 100 neurons). In other words, for a floating-point number divided by a variable, we already know that there can be differences in the conversion of a floating-point number, as previously mentioned in the “Compilation/Interpretation” section. The initialization of ${\bar{g}}_{syn}$ in the first python version was ${\bar{g}}_{syn}=0.036$, just a literal value. Thus, the python compiler in DFPP converts to a float value as 0.035999999999999997280, while C/C++ needs a previous mathematical operation, therefore ${\bar{g}}_{syn}=3.6/nNeurons,$ and in DFPP the compiler converts to ${\bar{g}}_{syn}=3.600000000000000088818/100.00000000000\;$and finally to ${\bar{g}}_{syn}=\;0.036000000000000004219$.

The final value is different among languages because the intermediate representations are different, and the operands must be converted so that they have the same exponent to perform division (or other mathematical operations in the floating-point unit), which can already cause different values. Then, rounding and normalization are done so that the number found is in the standard and size of the floating-point representation being used (IEEE 754). Therefore, we decided to directly initialize the variables with the literal value (3.6 in this case, check variable *p*_*g*_*syn*_ line 56 in SimulationParameters file); after this, the actual ${\bar{g}}_{syn}$ value is calculated in line 62 of *SimulationInitialization.h* file.

High floating-point precision (HFPP) variables deserved special attention. These variables must not be initialized with literal real numbers, as the compiler/interpreter converts the literal to the internal low precision representation (single or double precision) before assignment. For example, if we assign the literal 0.1 to an HFFP variable, the compiler/interpreter will transform that value into the closest possible value in the representation used (0.1000000000000000055511151231257827021182, in double precision) before the assignment. High-precision types, such as python's decimal or boost C/C++'s cpp_dec_float, normally allow variables to be initialized by string, preventing the compiler/interpreter from doing the conversion described above and allowing to initialize high-precision variables with exact values. To avoid this undue truncation, it was opted to directly initialize the variables with character type (String for Python and Char for C/C++), which enabled truncating the initial values and created a code hybrid structure that works with DFPP and HFPP.

Following the same logic, attributing floating-point variables and operations that return values in this format to high precision variables should be avoided because this would be inserting the same representation and rounding errors as floating-point variables in the high precision variable. The ideal situation is to use only highly accurate variables and compatible operations throughout the source code. Technically, the combination of an integer variable with a high precision variable does not cause a problem because the representation and conversion of an integer variable is accurate. Still, it was decided to initialize these variables as a character type.

Differently, Matlab does not allow this type of variable initialization as character with the standard floating-point type, which means that all operations in MATLAB were performed in double-precision arithmetic conforming to the IEEE standard 754.

#### Execution Order of the Instructions

As previously discussed, floating-point operations are not associative; hence, the order in which consecutive operations are performed changes the result. It must be ensured that all versions of the source code in the different languages/software programs used carry out the operations in the same order as the data. For example, the first versions of this code utilized different implementations of RK4 solver for each language/software, and thus the mathematical operations were performed in different orders. Therefore, an RK4 solver equal for all languages/software programs was chosen.

#### Using Libraries and/or Third-Party Source Code

Using libraries can generate errors in several different ways and represent a difficult problem to be solved, as we usually do not have access to their source. A library could implement the same function differently on different operating systems; hence, replicability is not achieved even using the same version of a library. For example, Blanco et al. (2020) utilized the native RK4 solver from Boost library. Herein, we opted for another approach as previously mentioned; therefore, the result for HFPP in Blanco et al. (2020) is different from the outcomes from this present study as shown in the next section. There is no guarantee that the operations follow the same execution order on different operating systems and ensure that the type conversions within a library's functions are the same across different operating systems/architecture. This situation was observed even when using the core standard libraries of the same programing and same version. The simulations presented in this study use two functions implemented by libraries: *power* and *exponential*. Although these functions are approved by the IEEE 754 and have large use in programming languages, we are aware of the undue precision error. As the experiments of this article were performed on the same machine using the same libraries in the same versions and only changing the operating system (Ubuntu Linux and Windows), we are led to believe that the error was due to some interference from the operating system. However, outside simple tests were performed with our own implementation of power function and in which we observed that the error does not appear in the different OS. Therefore, the error must be caused by implementation differences in the math library on different OSs, even though the libraries belong to the same versions. The floating-point library's source code is extensive and the order in which the internal functions are called is difficult to debug; thus, it was not possible to find the exact reason for this error in the library. The difference in results between Ubuntu Linux and Windows are regarding with machine epsilon (last bit 52nd) and it only happens in some cases. Finally, it was decided to utilize the language native functions due to two main facts: (1) no open-source code was found to replace these functions for the languages/software; and (2) reimplementing both functions were considered out of the scope of this study.

#### Porting the Code to Others Programing Languages/Software Programs

All the problems mentioned above can happen easier when using different languages. Two libraries/functions from different languages/software programs that are developed to execute the same tasks can have completely different implementations. In our case, we were using different libraries for C/C++ and Python languages which generated different results [For example *exp()* and *pow()* functions], without mentioning Matlab's own functions.

It is only possible to get around this problem by implementing our own functions or having access to the function's source code to ensure that the same functions, operations' order and data type will be regarded for the code execution. When implementing the function, we must be aware of the problems mentioned above, as well as the specific features of the languages/software program. For example, we can try to take advantage of simplified python operations on lists to implement a function quickly and simply. However, these operations can generate a different result from the implementation in another language such as C/C++ or Matlab software. Hence, sometimes it is a good strategy to give up the advantages offered by programing languages/software and write the code in a more verbose way to obtain the same results across languages/software programs.

#### Output

The data resulted from simulations went through a standardization process in order to facilitate the data organization and manipulation. Functions among all programing languages/software programs were created to properly store the outcomes in a pattern format (Check lines 134 and 185 from HH_BBT2020_allP file). The simulation outcomes have the information in their nomenclature to replicate the experiment as follows: the time step (*dt*), proportions of excitatory (*Ne)* and inhibitory (*Ni*) neurons, the reversal potential (*vl*), the biological simulation time in milliseconds (*t*), the precision utilized and the *Iapp* commutation. XPP/XPPAUT do not allow this configuration, thus the outcomes were stored manually through the software interface at the end of each simulation.

### Simulations Using High Floating-Point Precision (HFPP)

Carefully following the requirements just mentioned, the C/C++ refactored code was converted into Python and Matlab languages. Although there are differences among these language syntaxes, a careful translation was carried out to match them line-by-line, allowing more control over the manipulation, initialization of variables, functions, solver and code flow. Since the Matlab's implementation using HFPP was not executed (see Method section), the first set of simulations were those using HFPP in C/C++ and Python; resulting in a total of 24 scenarios (similar as shown in Figure 2, but for C/C++ and Python).

**Figure 3:**
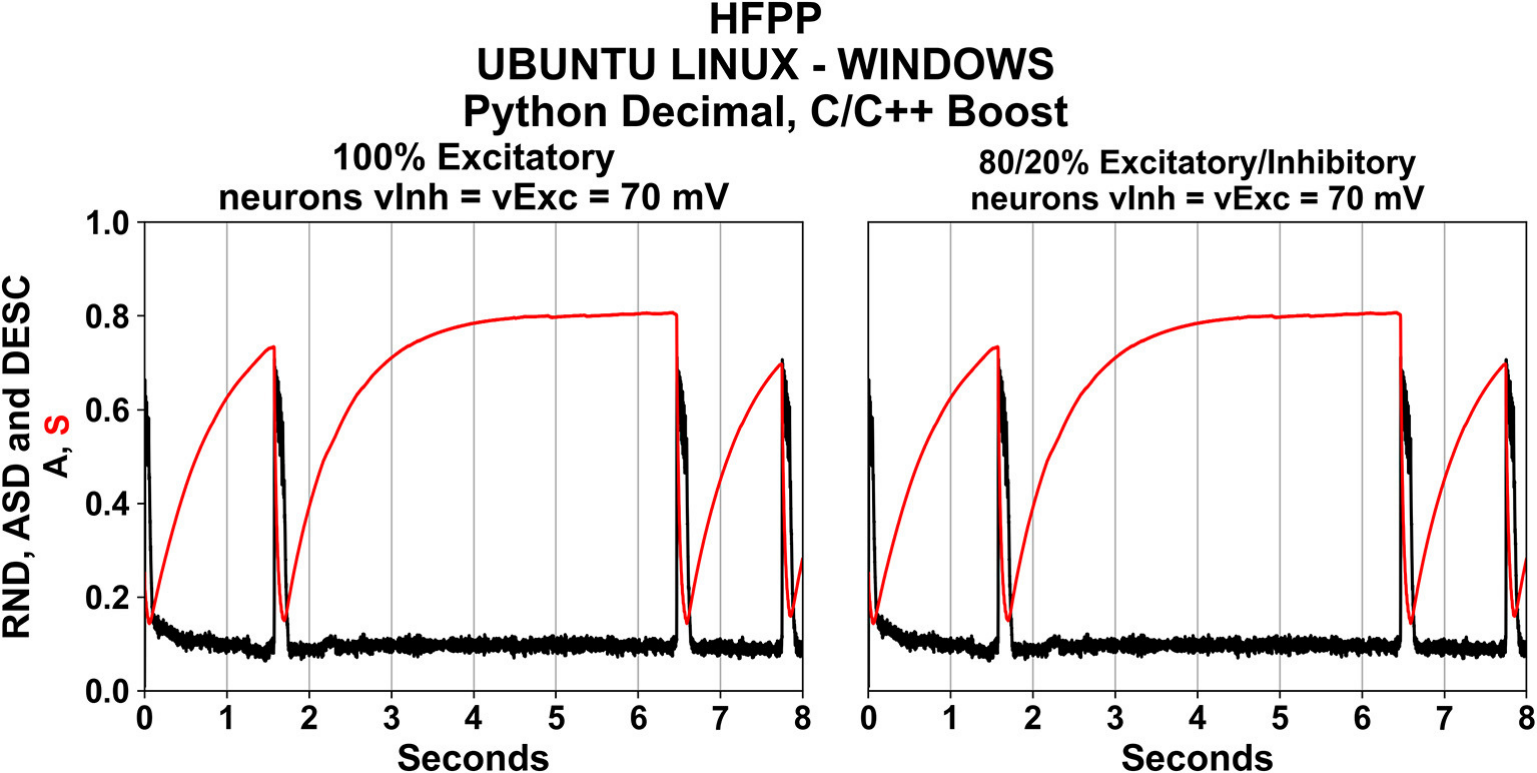
Using HFPP guaranteed the numerical replicability among simulations for different OSs and Languages.

The spontaneous network activity <A> and the network synaptic efficacy <S> have precisely the same values as expected (Figure 3). The simulations demanded large computational time and power as already presented in Table 5. Nevertheless, replicability was achieved during 8 s of biological simulation. These results will be compared with the rest of simulations using the absolute error equation (see Simulation Scenarios Section).

Although it is not fair to compare these results with XPP/XPPAUT due to its low precision, it was visually noticed that the first activity episode <A> in all the 12 simulations under XPP/XPPAUT started right before 2 s and ended right after the 2 s (Figure 2). Then with simulations using HFPP, the first episode started and ended before the 2 s (Figure 3).

### Simulations Using Default Float-Point Precision (DFPP)

**Figure 4:**
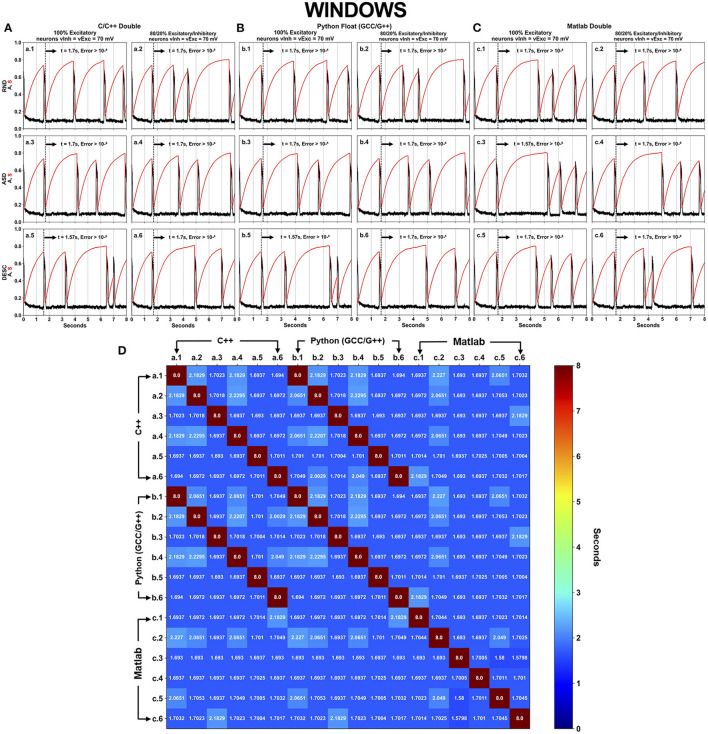
Neural activity results simulated using DFPP on Windows, which did not replicate the HFPP results. Each panel **(A–C)** has six (6–SPLT × SORT−2 × 3) scenarios explained in Figure 1 for C/C++, Python GCC/G++ and Matlab, respectively. The spontaneous network activity <A> and the network synaptic efficacy <S> are represented by the black and red curve profiles, respectively. The black vertical dashed line in each graph shows the time when the absolute error is >10^−6^ in comparison with the simulation that used HFPP (Figure 3). The last panel **(D)** displays a square matrix with an all-to-all comparison of these 18 scenarios based on the absolute error among simulations. Each cell represents the first moment in time (seconds) when the absolute error reached a value >10^−6^. Only the corresponding graphs of C/C++ (A.n panel) and Python GCC/G++ (B.n panel) replicated the results of <A> and <S> values. Simulations implemented with Matlab did not replicate the values as C/C++ and Python.

**Figure 5:**
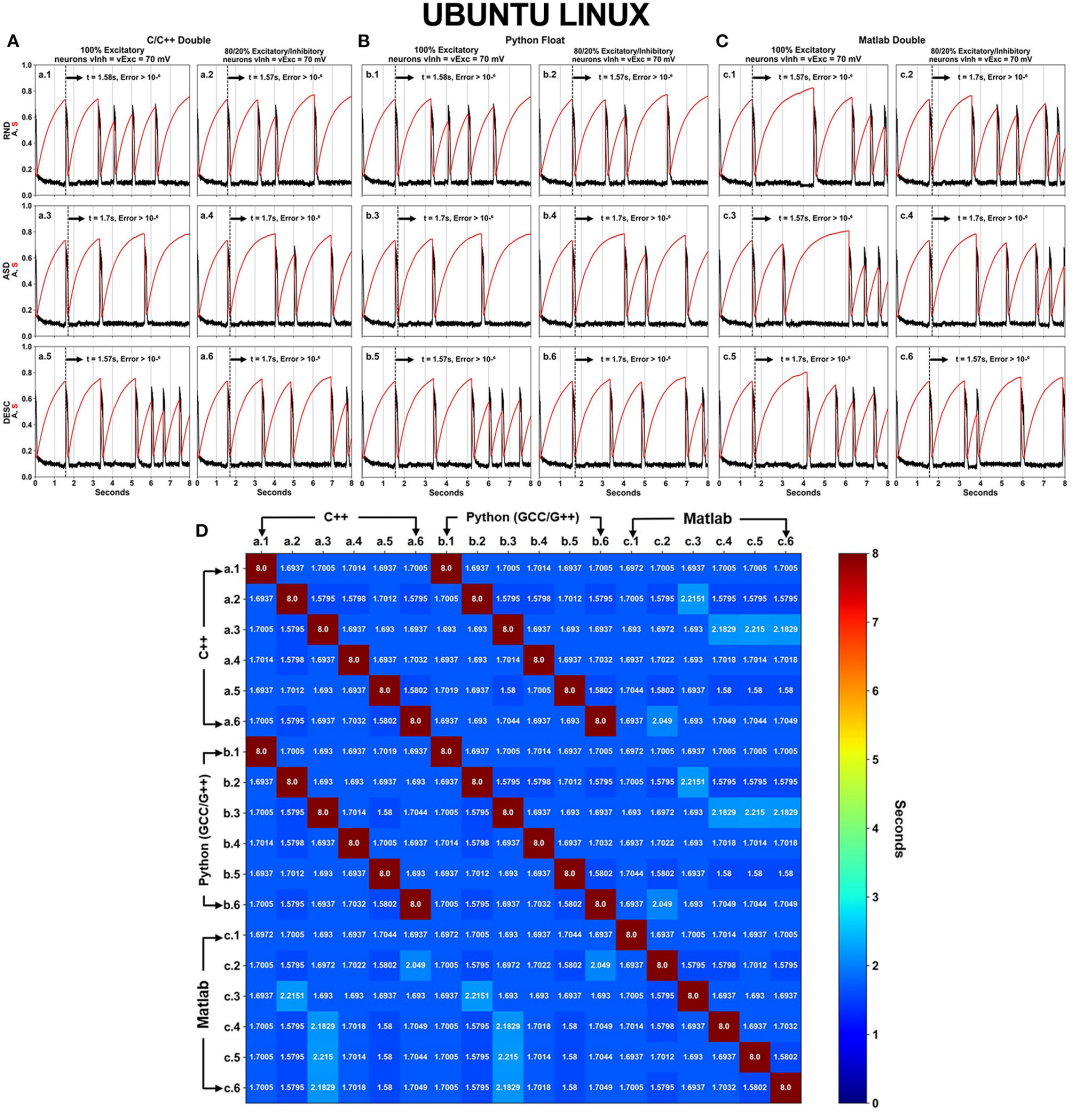
Neural activity results simulated using DFPP on Ubuntu Linux which did not replicate the HFPP results. Like in Figure 4, Panels **(A–C)** show the spontaneous network activity <A> (black curve) and the network synaptic efficacy <S> (red curve) for languages/software programs. The black vertical dashed line in each graph shows the time when the absolute error is >10^−6^ in comparison with the simulation that used HFPP (Figure 3). When the comparison was done among these 18 scenarios, replicability between C/C++ **(A)** and Python GCC/G++ **(B)** were observed as shown by dark red cells in **(D)**.

DFPP was performed under C/C++, Python and Matlab languages with their respective standard precision type (Table 3). The executed scenarios followed the same aspects as shown in Figure 1 (SPLT X SORT = 2 × 3 = 6), however now including the 3 languages/software programs, totaling 18 simulations for each OS (Figures 4, 5).

Python language is native in Ubuntu Linux OS, and the interpreter was compiled with the same version of GCC/G++ that was used to run the C/C++ simulations. On the other hand, Python and C/C++ languages had to be downloaded on Windows OS, and carefully installed with the same interpreter (GCC/G++) to perform the simulations. This process is not usual for Python language; therefore, the simulation with this configuration will be referred to throughout the text as “Python GCC/G++”. In addition, we understand that the most common way to use Python language on Windows OS is to download it directly from the official webpage or from the Microsoft Store. Therefore, we also performed the simulations with this conventional Python (Supplementary Figure 1) using the same version as Python GCC/G++ (v.3.8.0). The Matlab software program does not have this type of configuration in any OS, so the installation and performance of simulations was traditional.

Even with refactorization work done on the original code in C/C++ and converted to other programing languages/software, simulations using DFPP on Windows OS did not replicate the results (Figure 4) when compared with the simulation using HFPP (Figure 3). The dashed vertical line appeared before the 2 s of all simulations, signaling that the absolute error reached values > 10^−6^. However, we were expecting that error propagations were the same and simulations could be replicated among them. Only 6 cases between C/C++ and Python GCC/G++ replicated the results when the simulated model was equal, i.e., maintaining the same network configuration (SPLT and SORT). The network activity <A> and network synaptic efficacy <S> are identical in these scenarios [Figure 4D, cells (a.1 vs. b.1), (a.2 vs. b.2), …, (a.6 vs. b.6)]. The absolute error of network activity <A> for all the other simulations reached values >10^−6^ before the 3 s (Figure 4D, cells with blue hue).

The same source code across the languages was executed under Ubuntu Linux OS (Figure 5). Again, no replicability was achieved in any simulation when compared with the simulation using HFPP or compared with DFPP across the OSs. However, replicability did occur on the same six cases previously just shown (between C/C++ and Python) [Figure 5D cells (a.1 vs. b.1), (a.2 vs. b.2), …, (a.6 vs. b.6)]. Unfortunately, none of the simulations implemented on Matlab replicated across other languages.

The results of 36 simulations shown in Figures 4, 5 revealed that all of the scenarios using DFPP were unable to achieve replicability when compared with the simulations using HFPP. The vertical dashed lines in Figures 4A–C, 5A–C represent when the absolute error of <A> was >10^−6^; and the lines for all of them appeared around the end of the first activity episode, before the first 2 s. When the results were compared among simulations only using DFPP (Figures 4D, 5D), the lowest time when the absolute error reached >10^−6^ was 1.57948s (Figures 4D, 5D, cells in darker blue) and followed by values up to 2.2151s (Figure 4D, cells in cyan). After this value, only the maximum possible value of 8s was found (Figures 4D, 5D, cells in dark red); as previously mentioned, this means that identical values were achieved; hence, no absolute error was found in the 8s of simulation. There was no replicability among languages across OSs (Supplementary Figure 2).

### Analyzing the Sensitivity of Our Numerical Algorithm

Although it is not our main goal to cover the sensitivity analysis of our system, it is a point that has always been taken into account to separate whether unreliable results/behavior of the system are caused by the sensitivity to small perturbations or for the floating-point arithmetic. Therefore, Fuzzy tool (https://github.com/verificarlo/fuzzy), an adaptation of Verificarlo (Denis et al., 2015) to the Python language, was installed on Ubuntu Linux for Python language to carry out this task.

In turn, 6 simulations were executed under the Fuzzy environment for each of the 6 scenarios presented in panel B of Figure 5, for a total of 36 simulations (6 x {b1, b2, b3, b4, b5, b6}). Supplementary Figure 3 graphically shows only two of them. Under RND order of *Iapp* the scenarios are: (1) the network composed by 100% of excitatory neurons (b1), and (2) the network composed by 80/20% of excitatory/inhibitory neurons, respectively (b2). The six simulations reproduced the cycle neural activity, with high activity episodes followed by quiescence activity intervals. We did not observe any tendency of the system in going to unexpected states. As shown in all simulations executed in this study, after around 2 s of simulation the activity episodes appeared in different time moments affecting the mean and variance values (Supplementary Figure 3B). In this context, we checked how the perturbations could have affected the variance during the first 2 s of simulation, in which the mean and variance seen to not be visually perturbed (Supplementary Figure 3C). We calculated the “error” (subtraction with the original simulation presented in panel B of Figure 5) introduced by the fussy perturbation at every time step (6 × 200,000 time points, dt = 0.01 ms) for <A> and <S>. The distribution shapes of these values are shown in panel D in last graphs to the right. When the network was composed by 100% of excitatory neurons, the average activity <A> obtained a Mean = −2.7816e-10, Min = −3.4056e-05 and Max 4.1825e-05; and the synaptic efficacy <S> obtained a Mean = 1.5504e-09, Min = −1.5508e-06 and Max = 1.1021e-06. When the network was composed by 80/20% of excitatory/inhibitory neurons, respectively, the average activity <A> obtained a Mean = 6.9614e-10, Min = −4.4780e-05 and Max 5.4846e-05; and the synaptic efficacy <S> obtained a Mean = 2.0345e-10, Min = −2.0387e-06 and Max = 1.4448e-06. The smalls perturbations introduced by the Fuzzy tool did not cause unreliable results, even during the first 2 s of the simulation, where the two high activity episodes observed are happening at exactly the same time for all the simulations.

## Discussion

This study presented the numerical results of the activity of 100 neurons all-to-all coupling during early development. The results indicated that even using the same source code executed under the same hardware platform is not sufficient to achieve replicability (Table 6). The discrete and finite representation of floating-point numbers by using DFPP altered the results of mathematical operations. In turn, a summation of presynaptic activity conductances (6 scenarios shown in Figure 1) was used to prove that associativity and commutativity of these values led to different results. All the results using DFPP (Figures 4, 5 and Supplementary Figure 1) showed similar activity profiles (episodic bursts of intense activity separated by quiescent periods), however none of them replicated the simulations using HFPP (Figure 3 and Table 6, green check marks).

**Table 6:** Results summarizing table.

**Operating Systems →**	**Windows**					**Ubuntu Linux**			
**Precisions →**	**DFPP**		**HFPP**		**SORT**	**DFPP**		**HFPP**	
**Neurons proportions** ** → Languages/Software** **↓**	**100**	**80/20**	**100**	**80/20**	**↓**	**100**	**80/20**	**100**	**80/20**
XPP/XPPAUT (Version 6.9.0.31)	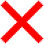	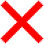	-	-	RND	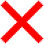	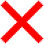	-	-
	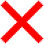	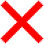	-	-	ASD	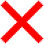	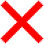	-	-
	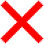	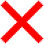	-	-	DESC	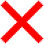	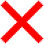	-	-
C/C++ (Version 9.3.0)	**WC**_**a.1**_	**WC**_**a.2**_	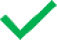	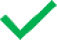	RND	**UC**_**a.1**_	**UC**_**a.2**_	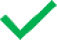	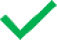
	**WC**_**a.3**_	**WC**_**a.4**_	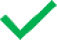	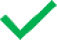	ASD	**UC**_**a.3**_	**UC**_**a.4**_	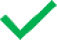	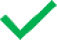
	**WC**_**a.5**_	**WC**_**a.6**_	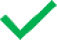	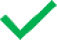	DESC	**UC**_**a.5**_	**UC**_**a.6**_	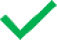	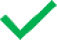
Python (Version 3.8.0)	**WP**_**b.1**_	**WP**_**b.2**_	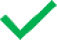	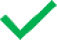	RND	**UP**_**b.1**_	**UP**_**b.2**_	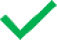	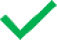
	**WP**_**b.3**_	**WP**_**b.4**_	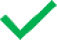	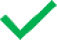	ASD	**UP**_**b.3**_	**UP**_**b.4**_	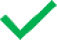	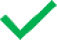
	**WP**_**b.5**_	**WP**_**b.6**_	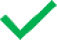	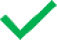	DESC	**UP**_**b.5**_	**UP**_**b.6**_	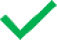	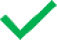
Matlab (Version 9.5.0 2017b)	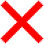	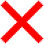	-	-	RND	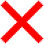	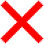	-	-
	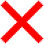	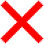	-	-	ASD	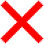	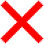	-	-
	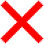	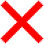	-	-	DESC	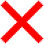	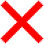	-	-

*Labels:* 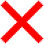 *No replicability; - Not executed;* 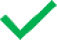 *Total replicability among simulations, only achieved by HFPP with C/C++ and Python languages in both OSs; **WC** simulations performed on Windows OS with C/C++ language that achieved replicability when the network configuration was identical with simulations in (**WP**) Windows OS with Python language [(**WC**_**a.1**_ vs. **WP**_**b.1**_), (**WC**_**a.2**_ vs. **WP**_**b.2**_), ... (**WC**_**a.6**_ vs. **WP**_**b.6**_)]; **UC** simulations performed on Ubuntu Linux OS with C/C++ language that achieved replicability when the network configuration was identical with simulations in (**UP**) Ubuntu Linux OS with Python language [(**UC**_**a.1**_ vs. **UP**_**b.1**_), (**UC**_**a.2**_ vs. **UP**_**b.2**_), ... (**UC**_**a.6**_ vs. **UP**_**b.6**_)]*.

The model was initially implemented under XPP/XPPAUT, a computational tool that offer benefits such as: a high-level script language and several useful and embedded functionalities. However, these benefits did not completely allow to control the way mathematical operations and functions (i.e., ODE's solver) are executed, which is a crucial point for replicability. This tool was not able to achieve replicability among simulations (Table 6, red X marks) and was not capable of performing simulations with HFPP (Table 6, black dash marks). In this context, the C/C++ source code was refactored and re-implemented under other program languages/software programs (Python and Matlab) and executed in Windows and Ubuntu Linux OSs.

In being aware that floating-point numbers are limitedly represented using DFPP, it is expected that error propagation would be the same across simulations; hence, the results should be replicated. Nevertheless, none of the results showed replicability across OSs (Supplementary Figure 2), suggesting that compilers (even the same version) behaved differently across OSs. Since all simulations were executed under the same HPs, the non-replicability observed across the OSs were indirectly caused by the programming language, source code, libraries being used (with their respective compiler and compilation options).

Fortunately, only six scenarios that used the same model parameters were replicated across languages (C/C++ and Python) under the same OS (Figures 4D, 5D, dark red cells and Table 6, WC (Windows—C/C++) and WP (Windows—Python) and UC (Ubuntu Linux—C/C++) and UP (Ubuntu Linux—Python). Two important aspects were crucial to achieve these replications: (1) the careful refactorization process applied in Blanco et al. (2020) of C/C++ source code and its translation to Python, allowing full control over variable initialization, the order of operations and the use of the same functions; and (2) the fact that Python binary source codes were built under the same C/C++ compiler. Unfortunately, we were not able to rebuild Matlab binary code; actually, we do not even know if this is possible, maybe due to commercial issues. In this case, replicability was not achieved in this software (Table 6, red X marks) and simulations with HFPP were not performed (Table 6, black dash marks).

A simple neural network case helped to conclude that numerical replicability could be achieved using HFPP; however, it needed specific third-party libraries and required high computational cost. Only 12 executed scenarios out of 36 (630 possible comparisons) using DFPP were replicated because a meticulous refactorization process was performed to the source code. The other scenarios did not replicate the results and errors appeared early. This situation could be worse if larger and more complex models are simulated, since more error sources will be introduced. In this context, it could be presumed that computational replicability is very difficult to achieve and needs people with profound computational expertise, mainly in the software development area. Finally, the research community, and not only fields related to neuroscience, must be aware that conducting analyses using numerical simulations which implement floating-point numbers operations can lead to different conclusions.

## Data Availability Statement

The original contributions presented in the study are included in the article/Supplementary Material, further inquiries can be directed to the corresponding author/s.

## Author Contributions

PL and WB contributed to the study design, computer simulations, and writing of the article. BO and AS contributed to refactoring of source code, computer simulations, and writing of the article. All authors contributed to the article and approved the submitted version.

## Funding

This research started in 2016 when WB was hosted by the Mathematics department and the Institute of Molecular Biophysics at Florida State University. WB was also supported by a scholarship (Process #202320/2015-4) from the Brazilian National Council for Scientific and Technological Development (*Conselho Nacional de Desenvolvimento Cient*í*fico e Tecnológico—CNPq*—https://www.gov.br/cnpq/pt-br). PL was supported by an undergraduate scientific research scholarship from CNPq in 2017, from the State University of Rio Grande do Norte (UERN—http://portal.uern.br/) in 2018 and graduate scholarship from CAPES (*Coordenação de Aperfeiçoamento de Pessoal de N*í*vel Superior*—https://www.gov.br/capes/pt-br) during his master's degree at Postgraduate Program in Bioinformatics at Federal University of Rio Grande do Norte in 2020. This study was financed in part by the Coordenação de Aperfeiçoamento de Pessoal de Nível Superior - Brasil (CAPES) - Finance Code 001.

## Conflict of Interest

The authors declare that the research was conducted in the absence of any commercial or financial relationships that could be construed as a potential conflict of interest.

## Publisher's Note

All claims expressed in this article are solely those of the authors and do not necessarily represent those of their affiliated organizations, or those of the publisher, the editors and the reviewers. Any product that may be evaluated in this article, or claim that may be made by its manufacturer, is not guaranteed or endorsed by the publisher.
