# Supplementary material for: Mitigating Computer Limitations in Replicating Numerical Simulations of a Neural Network Model With Hodgkin-Huxley-Type Neurons
Source: Front Neuroinform. 2022 May 12;16:874234. doi: 10.3389/fninf.2022.874234 (PMC9135410; doi:10.3389/fninf.2022.874234)
Supplement: Supplementary file 1 [file Data_Sheet_1.pdf]

Supporting information

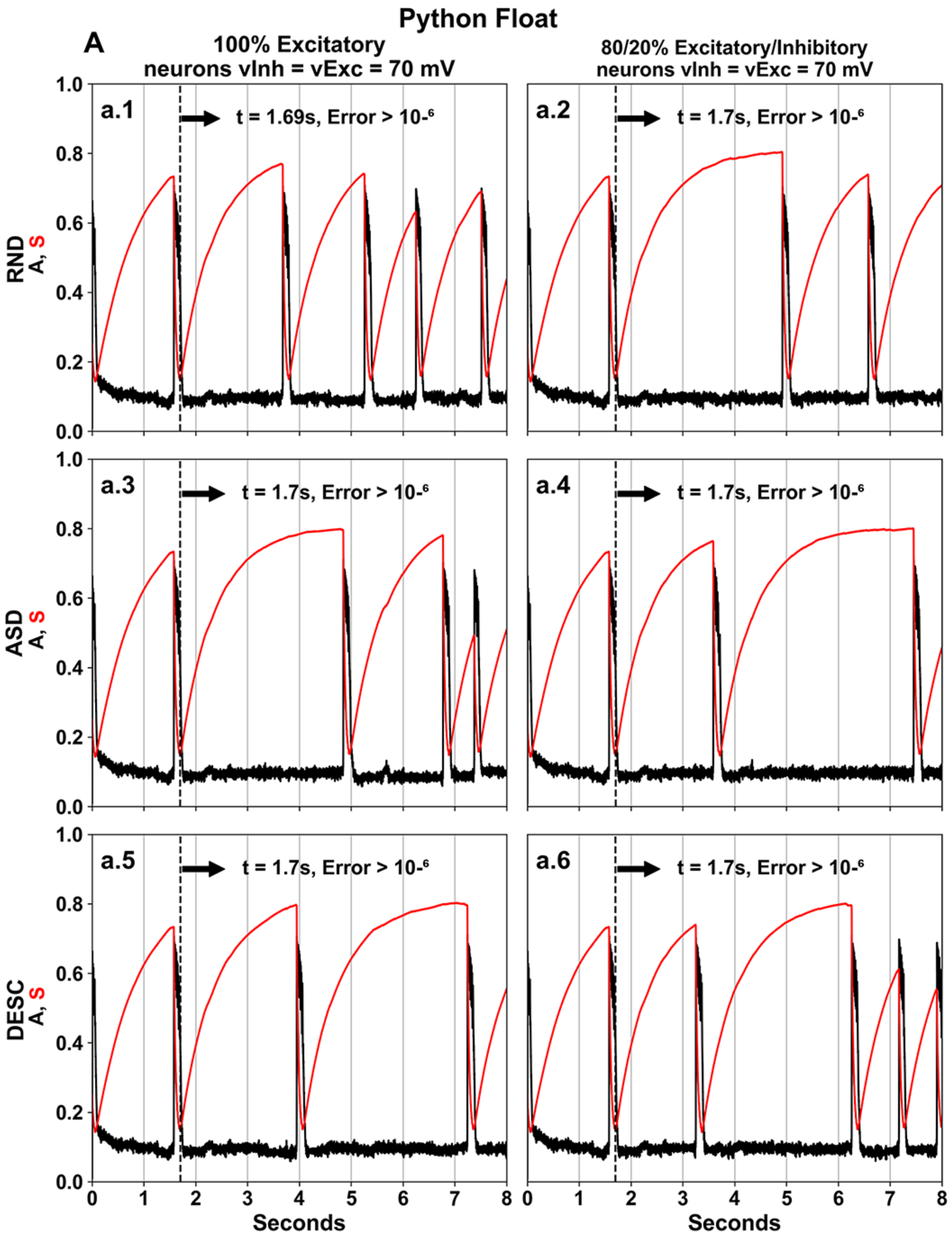

Supplementary Figure 1. Python simulations using DFPP (float) under Windows OS.

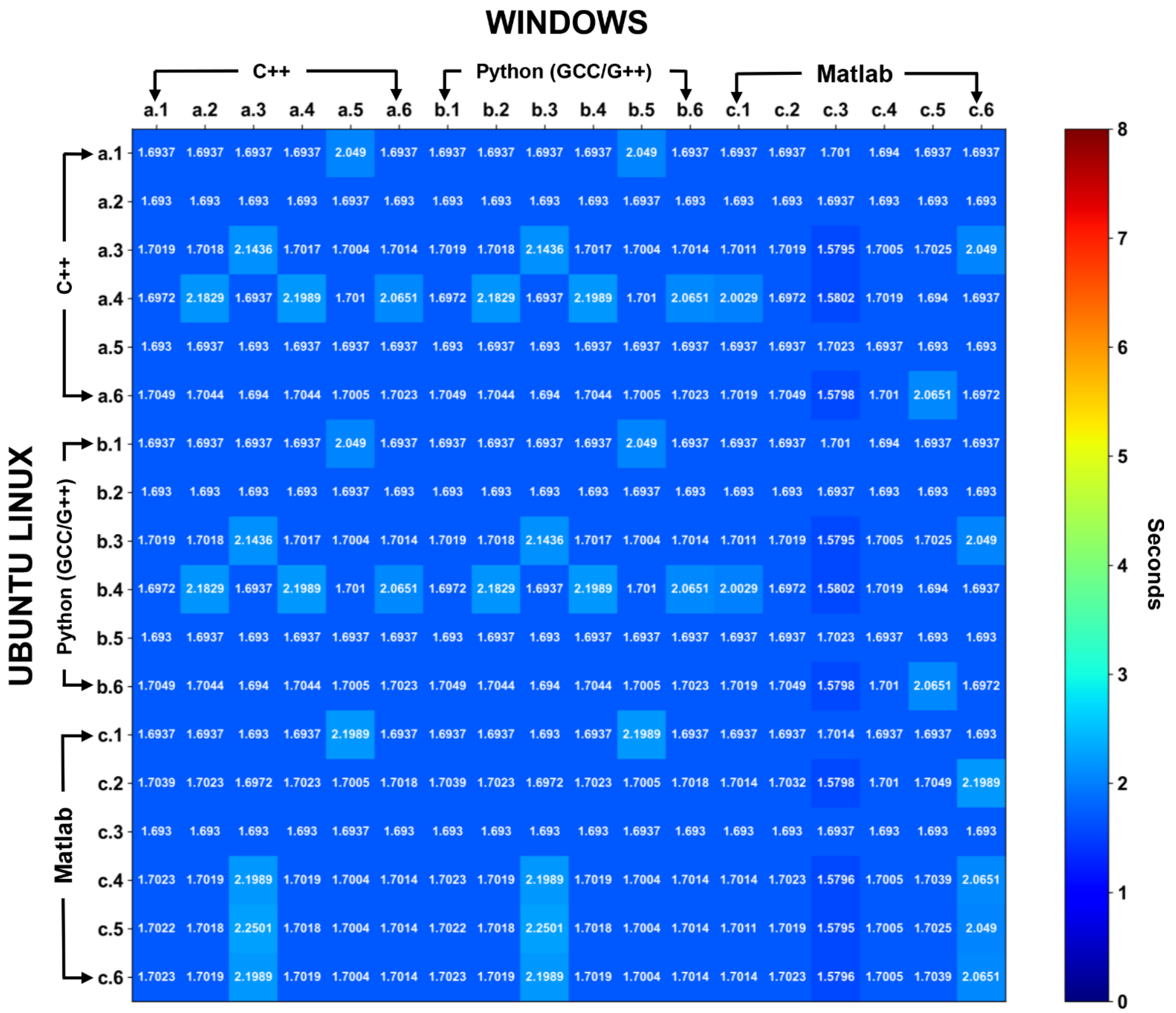

Supplementary Figure 2. Comparing simulations using DFPP among programing languages across operating systems (Ubuntu Linux and Windows). Each table cell represents the moment (seconds) in time when the absolute error reached the value > 10^{-6}.

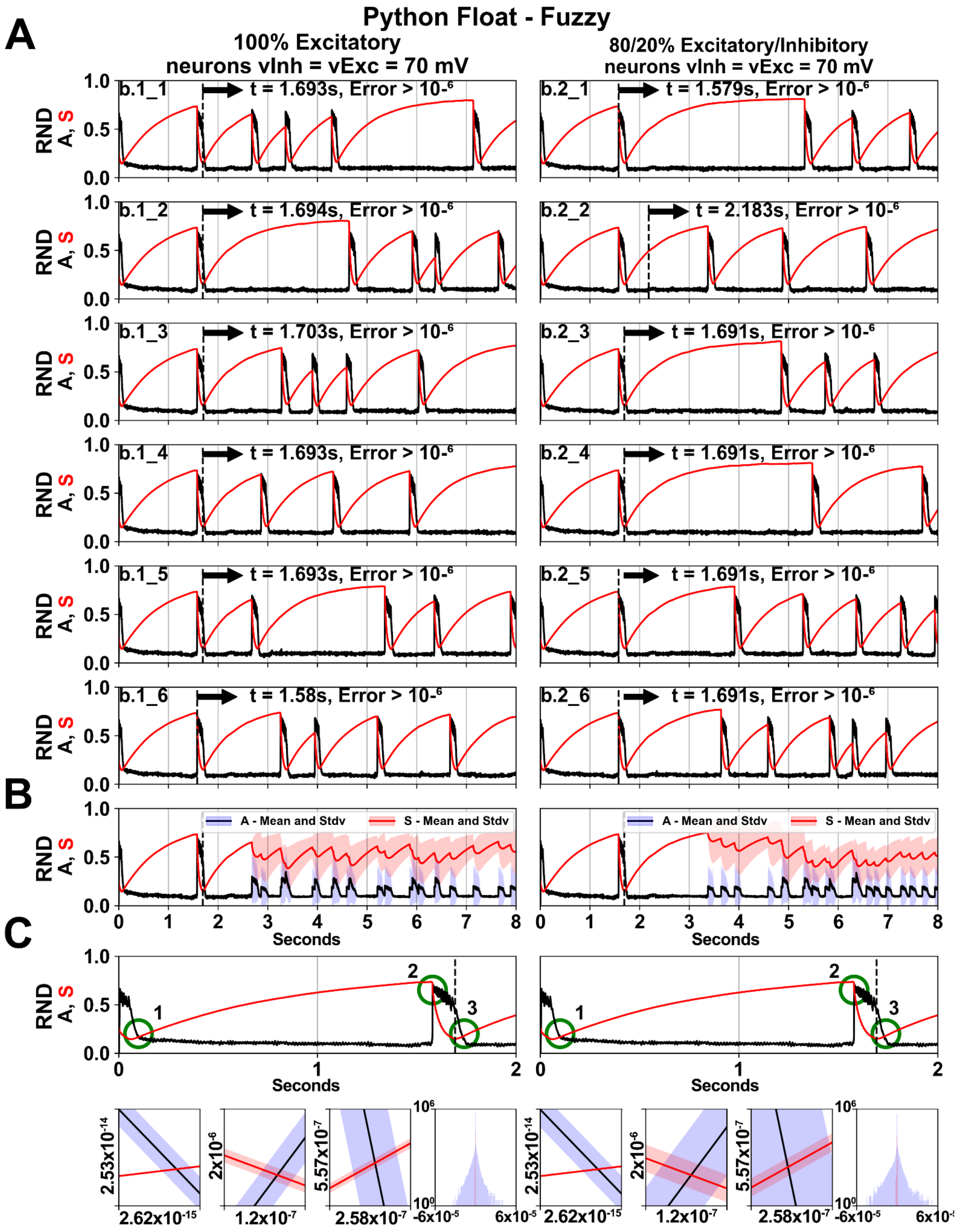

Supplementary Figure 3. **Simulations executed under the Fuzzy environment for Python did not show unreliable results.** 6 simulations were executed for the 6 scenarios presented in panel B of **Figure 5**, for a total of 36 simulations (6 x {b.1, b.2, b.3, b.4, b.5, b.6}). A) Only two of them are shown, for a total of 12 simulations. Under RND order of *Iapp* the scenarios are: First column, the network composed by 100% of excitatory neurons (b.1), and second column, the network composed by 80/20% of excitatory/inhibitory neurons, respectively (b.2). After around 2s, activity episodes appeared in different time moments for all

the 12 simulations, affecting the mean and variance values (panel B). C) We checked how the perturbations could have affected the variance during the first 2s of simulation, in which the mean and variance seen to not be visually perturbed (first row). Three graphs, with a very small time-window, were selected to graphically represent the mean ± std (second row). We calculated the “error” (subtraction with the original simulation presented in panel B of **Figure 5**) introduced by the fussy perturbation at every time step (6 x 200 000 time points, dt=0.01ms) for <A> and <S>. The distribution shapes of these values are shown in last graphs to the right.
